# A nomogram for predicting in‐hospital death in a multinational cohort of patients with takotsubo syndrome

**DOI:** 10.1111/eci.70190

**Published:** 2026-03-26

**Authors:** Yuyi Chen, Amanda Chang, Fangyuan Cheng, Davide Di Vece, Michael Würdinger, Philipp Theil, Tou Kun Chong, Christian Templin, Jian Chen, Xiaodong Wu, Kan Liu

**Affiliations:** ^1^ Division of Cardiology Washington University in St. Louis Saint Louis Missouri USA; ^2^ Department of Internal Medicine University of Iowa Iowa City Iowa USA; ^3^ Department of Ultrasound The Fifth Affiliated Hospital of Sun Yat‐Sen University Zhuhai Guangdong China; ^4^ Department of Cardiology and Internal Medicine B University Medicine Greifswald Greifswald Germany; ^5^ DZHK (German Centre for Cardiovascular Research) Partner Site Greifswald Greifswald Germany; ^6^ First Clinic of Internal Medicine, Department of Internal Medicine University of Genoa Genoa Italy; ^7^ Department of Cardiology University Heart Center, University Hospital Zurich, and University of Zurich Zurich Switzerland; ^8^ Department of Cardiology Kiang Wu Hospital Macao Special Administrative Region of the People's Republic of China China; ^9^ Center for Molecular Cardiology, Schlieren Campus University of Zurich Zurich Switzerland; ^10^ Swiss CardioVascularClinic Private Hospital Bethanien Zurich Switzerland; ^11^ Department of Cardiology The Fifth Affiliated Hospital of Sun Yat‐Sen University Zhuhai Guangdong China; ^12^ Department of Electrical and Computer Engineering University of Iowa Iowa City Iowa USA

**Keywords:** in‐hospital death, nomogram, prognostic score, risk stratification, takotsubo syndrome

## Abstract

**Background:**

An effective risk stratification model on hospitalized patients with takotsubo syndrome (TTS) helps guide treatment to mitigate adverse events and improve prognosis. We aimed to develop a nomogram for predicting in‐hospital death in a multinational cohort of TTS patients.

**Methods:**

We enrolled 829 TTS patients from AmSC Research Network, InterTAK registry and ChiTTS registry, classified into the training (*n* = 578), test (*n* = 145) and external validation (*n* = 106) cohorts.

**Results:**

Body mass index (BMI), chronic kidney disease (CKD), neurologic disorders, cardiogenic shock, low systolic blood pressure (SBP, <122 mmHg) and abnormal white blood cell (WBC, ≥11.3 × 10^9^/L) were independent positive predictors, while chest pain was an independent negative predictor of in‐hospital death. A nomogram was constructed to predict in‐hospital death in TTS patients based on these seven independent variables, which showed that the area under the curves (AUCs) in the training and test cohorts were .854 (95% CI: .805–.904, *p* < .001) and .836 (95% CI: .737–.934, *p* < .001), respectively. The calibration curves showed good consistency between the prediction of the nomogram and the actual observation in both the training and test cohorts. Decision curve analyses indicated that the use of the nomogram to predict in‐hospital death in TTS patients could provide better net benefit than the ‘treat all’ or ‘treat none’ strategies when the threshold probability ranged from 2% to 75% in the training cohort and from 2% to 72% in the test cohort. The nomogram was further validated, with AUC of .838 (95% CI: .663–1.000, *p* = .003) in the external validation cohort.

**Conclusions:**

The nomogram, composed of BMI, CKD, neurologic disorders, chest pain, cardiogenic shock, low SBP and abnormal WBC, helps predict in‐hospital death in TTS patients.

## Graphical abstract

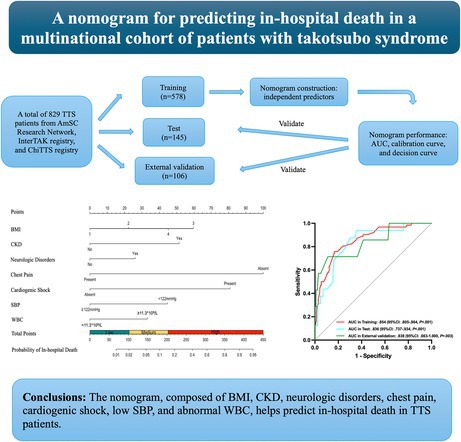

## 1 INTRODUCTION

Takotsubo syndrome (TTS) is an increasingly recognized cardiovascular disorder in hospitalized patients, characterized by left ventricular dysfunction without obstructive coronary artery disease.^1^, ^2^, ^3^, ^4^ Although initially considered a benign and fully reversible condition, TTS has been found to have heterogeneous phenotypes and courses. Evidence is accumulating that TTS patients have poor short‐ and long‐term outcomes.^5^, ^6^, ^7^ Of concern, a recent study indicated that TTS associated in‐hospital complications and mortality is on the rise.^8^ Risk stratification is particularly important for guiding decision‐making and treatment to mitigate adverse events and improve prognosis in hospitalized TTS patients. Although the major TTS registries have previously formed vital prognostic scores to predict short‐ and long‐term outcomes in TTS patients,^9^, ^10^ a nomogram model on a cohort of TTS patients with greater diversity and ethnicity has yet to be established. To build a widely applicable clinical prediction tool, we aimed to develop a nomogram for predicting in‐hospital death based on admission clinical data in TTS patients from three major national/international TTS registries and research network.

## 2 METHODS

### 2.1 Study population

A total of 829 TTS patients were enrolled from AmSC (American Stress Cardiomyopathy) Research Network (*n* = 527), InterTAK registry^11^ (*n* = 122) and ChiTTS registry^12^ (*n* = 180), diagnosed between November 2004 and July 2024. The AmSC Research Network is an observational and retrospective research study established among 10 university‐affiliated or regional referral medical centers inside the USA (Washington University in St. Louis, University of Iowa, State University of New York, University of North Carolina, Weill Cornell Medical College, Kansas University, Lankenau Medical Center, Northwest Health Medical Center, Providence Regional Medical Center and Saint Louis University). The AmSC Research Network was established to augment the machine learning algorithms using large‐scale multi‐institution and multi‐vendor echocardiographic datasets, as well as clinical data metrics to increase the robustness, generalizability and interpretability of prediction models in clinical and imaging diagnosis and prognosis of TTS patients.^13^, ^14^ In order to maintain consistency and completeness of clinical data collection and analysis in the present study, we only enrolled the patient data of AmSC Research Network in two major tertiary cardiovascular centers (Washington University in St. Louis and University of Iowa). TTS diagnosis was based on the InterTAK diagnostic criteria.^1^ This study complied with the principles contained in the Declaration of Helsinki and was approved by the institutional review board of Washington University in St. Louis. Data reporting follows the principles put forth by the STROBE initiative.

### 2.2 Data collection and primary endpoint event

Baseline clinical characteristics were recorded for every patient, including demographics (age, sex and race), medical history (based on prior diagnosis, including smoking, hypertension, diabetes mellitus, hyperlipidemia, atrial fibrillation, coronary artery disease, chronic obstructive pulmonary disease [COPD], asthma, chronic kidney disease [CKD], malignancy, psychiatric disorders, neurologic disorders [including ischemic stroke, intracerebral haemorrhage, migraine, epilepsy, seizures and so on] and acute neurologic disorders), triggering factor, symptoms (pre‐admission/admission, including chest pain and shortness of breath), presence of cardiogenic shock (pre‐admission/admission, based on ICD code/clinical diagnosis), vital signs (on admission, including body mass index [BMI], heart rate and systolic blood pressure [SBP]/diastolic blood pressure [DBP]), laboratory biomarkers (on admission, including troponin, white blood cell [WBC] and creatinine), electrocardiographic parameters (on admission, including ST‐segment elevation, ST‐segment depression, T‐wave inversion and QTc prolongation) and echocardiographic parameters (on admission, including type of TTS, left ventricular ejection fraction [LVEF] and right ventricular [RV] involvement). The primary endpoint event was in‐hospital all‐cause death. Seven hundred and twenty‐three TTS patients had raw echocardiographic imaging data and were randomly classified into the training (80%, *n* = 578) and test (20%, *n* = 145) cohorts based on in‐hospital death. Meanwhile, we enrolled 106 TTS patients between June 2012 and June 2024 from the ChiTTS registry as the external validation cohort, who did not have raw echocardiographic imaging data.

### 2.3 Statistical analysis

Data were presented with mean and standard deviation or median and interquartile range (IQR) for continuous variables and with number and percentage for categorical variables. Independent sample *t*‐test was used for comparisons of normal distributed data. Comparisons were performed with Mann Whitney *U* test for non‐normal distributed data. Categorical data were compared using Chi‐Square test. Restricted cubic spline curves were used to assess the relationship between several continuous variables (including BMI, heart rate, SBP and WBC) and the risk of primary endpoint event with 5 knots at the 5th, 35th, 50th, 65th and 95th percentiles of these variables. Multivariate logistic regression analysis was performed to select independent predictors of the primary endpoint event, and adjusted variables included those with *p* < .2 in univariate analyses and clinical meaning based on previous studies.^5^, ^9^, ^10^, ^15^, ^16^, ^17^ The new prognostic score model was presented with the nomogram, and the probability of in‐hospital death was estimated using the nomogram. Receiver operating characteristic (ROC) curves were performed to evaluate the discriminative performance of the nomogram. Calibration curves were used to assess the consistent ability between the predicted and actual probability of in‐hospital death. Decision curve analyses were performed to evaluate the clinical benefit and utility of the nomogram. To assess whether the nomogram would improve the discrimination when compared to previous prognostic scores, C‐index, net reclassification improvement (NRI) and integrated discrimination improvement (IDI) were calculated and compared. Missing values of all variables were <10% (Tables S1 and S2). Mean or median was used when a missing value was a continuous variable and mode was used when a missing value was a categorical variable. All analyses were performed with SPSS version 27 and R version 4.4.1. *p* Values <.05 were considered statistically significant.

## 3 RESULTS

A total of 829 TTS patients were enrolled, among which 578 were allocated to the training cohort, 145 to the test cohort and 106 to the external validation cohort. Except for race and QTc prolongation, all other features were comparable between the training and test cohorts (Table 1).

**TABLE 1:** Baseline clinical features and in‐hospital death between the training and test cohorts.

	Training (*n* = 578)	Test (*n* = 145)	*p*‐Value
Age, years	64.2 ± 15.3	65.1 ± 14.4	.522
Male, *n* (%)	115 (19.9)	29 (20.0)	.978
Race, *n* (%)			
White	419 (72.5)	121 (83.4)	.044
Black	63 (10.9)	8 (5.5)	
Asian	69 (11.9)	13 (9.0)	
Unknown	27 (4.7)	3 (2.1)	
BMI, kg/m^2^	26.4 ± 6.9	26.3 ± 6.3	.876
BMI, *n* (%)			
Underweight	46 (8.0)	15 (10.3)	.431
Normal weight	226 (39.1)	46 (31.7)	
Overweight	175 (30.3)	50 (34.5)	
Mild obesity	69 (11.9)	21 (14.5)	
Moderate obesity	34 (5.9)	9 (6.2)	
Severe obesity	28 (4.8)	4 (2.8)	
Current smoker, *n* (%)	126 (21.8)	37 (25.5)	.338
Hypertension, *n* (%)	353 (61.1)	82 (56.6)	.320
Diabetes mellitus, *n* (%)	136 (23.5)	30 (20.7)	.467
Hyperlipidemia, *n* (%)	267 (46.2)	62 (42.8)	.458
Atrial fibrillation, *n* (%)	85 (14.7)	21 (14.5)	.946
Coronary artery disease, *n* (%)	115 (19.9)	31 (21.4)	.691
COPD, *n* (%)	86 (14.9)	16 (11.0)	.234
Asthma, *n* (%)	36 (6.2)	13 (9.0)	.241
CKD, *n* (%)	93 (16.1)	21 (14.5)	.635
Malignancy, *n* (%)	138 (23.9)	46 (31.7)	.052
Psychiatric disorders, *n* (%)	219 (37.9)	52 (35.9)	.652
Neurologic disorders, *n* (%)	218 (37.7)	49 (33.8)	.381
Acute neurologic disorders, *n* (%)	107 (18.5)	27 (18.6)	.976
Physical trigger, *n* (%)	401 (69.4)	103 (71.0)	.698
Chest pain, *n* (%)	196 (33.9)	61 (42.1)	.066
Shortness of breath, *n* (%)	279 (48.3)	78 (53.8)	.234
Cardiogenic shock, *n* (%)	83 (14.4)	19 (13.1)	.698
Heart rate, bpm	95.8 ± 23.2	91.7 ± 20.5	.053
SBP, mmHg	123.9 ± 26.3	121.5 ± 23.2	.317
DBP, mmHg	75.5 ± 17.6	73.8 ± 15.5	.292
Troponin, ng/mL	.46 (.11, 1.98)	.60 (.13, 3.17)	.192
WBC, ×10^9^/L	12.4 ± 6.8	12.1 ± 6.9	.609
Creatinine, mg/dL	.9 (.7, 1.2)	.9 (.7, 1.2)	.471
ST‐segment elevation, *n* (%)	126 (21.8)	31 (21.4)	.913
ST‐segment depression, *n* (%)	92 (15.9)	25 (17.2)	.699
T‐wave inversion, *n* (%)	308 (53.3)	80 (55.2)	.684
QTc prolongation, *n* (%)	390 (67.5)	83 (57.2)	.021
Type of TTS, *n* (%)			
Apical	411 (72.6)	107 (75.4)	.762
Mid‐ventricular	42 (7.4)	13 (9.2)	
Basal	35 (6.2)	6 (4.2)	
Focal	38 (6.7)	8 (5.6)	
Global	40 (7.1)	8 (5.6)	
LVEF, %	37.9 ± 12.0	37.9 ± 11.9	.996
RV involvement, *n* (%)	108 (20.3)	20 (14.6)	.133
In‐hospital death, *n* (%)	62 (10.7)	16 (11.0)	.915

Abbreviations: BMI, body mass index; CKD, chronic kidney disease; COPD, chronic obstructive pulmonary disease; DBP, diastolic blood pressure; LVEF, left ventricular ejection fraction; RV, right ventricular; SBP, systolic blood pressure; TTS, takotsubo syndrome; WBC, white blood cell.

### 3.1 Independent predictors of in‐hospital death in TTS patients in the training cohort

Compared to patients who survived hospitalization, those who experienced in‐hospital death were more frequently to have underweight, obesity, CKD, physical trigger, cardiogenic shock and RV involvement, and less likely to present with chest pain. Additionally, they had lower SBP, DBP and LVEF, and higher WBC and creatinine (Table 2).

**TABLE 2:** Baseline clinical features of patients with and without in‐hospital death in the training cohort.

	In‐hospital death (*n* = 62)	No in‐hospital death (*n* = 516)	*p*‐Value
Age, years	63.7 ± 16.6	64.2 ± 15.2	.777
Male, *n* (%)	18 (29.0)	97 (18.8)	.057
Race, *n* (%)			
White	47 (75.8)	372 (72.1)	.886
Black	5 (8.1)	58 (11.2)	
Asian	7 (11.3)	62 (12.0)	
Unknown	3 (4.8)	24 (4.7)	
BMI, kg/m^2^	27.8 ± 9.5	26.2 ± 6.5	.215
BMI, *n* (%)			
Underweight	9 (14.5)	37 (7.2)	.001
Normal weight	20 (32.3)	206 (39.9)	
Overweight	11 (17.7)	164 (31.8)	
Mild obesity	9 (14.5)	60 (11.6)	
Moderate obesity	4 (6.5)	30 (5.8)	
Severe obesity	9 (14.5)	19 (3.7)	
Current smoker, *n* (%)	10 (16.1)	116 (22.5)	.252
Hypertension, *n* (%)	39 (62.9)	314 (60.9)	.754
Diabetes mellitus, *n* (%)	20 (32.3)	116 (22.5)	.086
Hyperlipidemia, *n* (%)	24 (38.7)	243 (47.1)	.211
Atrial fibrillation, *n* (%)	13 (21.0)	72 (14.0)	.141
Coronary artery disease, *n* (%)	11 (17.7)	104 (20.2)	.653
COPD, *n* (%)	9 (14.5)	77 (14.9)	.932
Asthma, *n* (%)	1 (1.6)	35 (6.8)	.189
CKD, *n* (%)	19 (30.6)	74 (14.3)	.001
Malignancy, *n* (%)	20 (32.3)	118 (22.9)	.101
Psychiatric disorders, *n* (%)	21 (33.9)	198 (38.4)	.490
Neurologic disorders, *n* (%)	29 (46.8)	189 (36.6)	.119
Acute neurologic disorders, *n* (%)	17 (27.4)	90 (17.4)	.056
Physical trigger, *n* (%)	55 (88.7)	346 (67.1)	<.001
Chest pain, *n* (%)	4 (6.5)	192 (37.2)	<.001
Shortness of breath, *n* (%)	33 (53.2)	246 (47.7)	.409
Cardiogenic shock, *n* (%)	28 (45.2)	55 (10.7)	<.001
Heart rate, bpm	99.6 ± 22.6	95.3 ± 23.2	.167
SBP, mmHg	110.6 ± 22.6	125.4 ± 26.3	<.001
DBP, mmHg	68.1 ± 16.8	76.3 ± 17.5	<.001
Troponin, ng/mL	.38 (.05, 1.61)	.46 (.12, 2.04)	.334
WBC, ×10^9^/L	15.6 ± 9.4	12.0 ± 6.3	.005
Creatinine, mg/dL	1.1 (.8, 1.6)	.9 (.7, 1.1)	.004
ST‐segment elevation, *n* (%)	12 (19.4)	114 (22.1)	.622
ST‐segment depression, *n* (%)	15 (24.2)	77 (14.9)	.059
T‐wave inversion, *n* (%)	29 (46.8)	279 (54.1)	.277
QTc prolongation, *n* (%)	37 (59.7)	353 (68.4)	.165
Type of TTS, *n* (%)			
Apical	49 (79.0)	370 (71.7)	.358
Mid‐ventricular	2 (3.2)	41 (7.9)	
Basal	3 (4.8)	34 (6.6)	
Focal	2 (3.2)	37 (7.2)	
Global	6 (9.7)	34 (6.6)	
LVEF, %	34.6 ± 11.5	38.3 ± 12.0	.019
RV involvement, *n* (%)	19 (30.6)	102 (19.8)	.047

Abbreviations: BMI, body mass index; CKD, chronic kidney disease; COPD, chronic obstructive pulmonary disease; DBP, diastolic blood pressure; LVEF, left ventricular ejection fraction; RV, right ventricular; SBP, systolic blood pressure; TTS, takotsubo syndrome; WBC, white blood cell.

Univariate logistic regression analysis showed that patients with severe obesity or underweight had a higher risk of in‐hospital death compared to those with normal weight. The risk of in‐hospital death was similar between mild and moderate obesity. The risk of in‐hospital death in patients with overweight was slightly lower than those with normal weight, but it did not reach statistical significance (Table S3). Furthermore, restricted cubic spline curve showed that the risk of in‐hospital death was similar between normal weight and overweight (Figure S1A). Therefore, patients were categorized into four groups for multivariate analysis: normal weight/overweight, mild‐to‐moderate obesity, severe obesity and underweight. Additionally, restricted cubic spline curves revealed that the risk of in‐hospital death increased when heart rate was >95 bpm (Figure S1B), the risk of in‐hospital death increased with the decrease of SBP levels when SBP was <122 mmHg (Figure S1C), and the risk of in‐hospital death increased with the increase of WBC counts when WBC was ≥11.3 × 10^9^/L (Figure S1D).

Adjusted for age ≥65 years, sex, race, BMI, diabetes mellitus, atrial fibrillation, asthma, CKD, neurologic disorders, malignancy, physical trigger, chest pain, cardiogenic shock, heart rate ≥95 bpm, SBP <122 mmHg, WBC ≥11.3 × 10^9^/L, ST‐segment elevation, ST‐segment depression, QTc prolongation, LVEF ≤35%, and RV involvement, multivariate logistic regression analysis showed that BMI (mild‐to‐moderate obesity vs. normal weight/overweight: adjusted OR 2.18, 95% CI: .90–5.29, *p* = .085; severe obesity vs. normal weight/overweight: adjusted OR 3.66, 95% CI: 1.13–11.88, *p* = .031; underweight vs. normal weight/overweight: adjusted OR 3.57, 95% CI: 1.27–10.05, *p* = .016), CKD (adjusted OR 2.73, 95% CI: 1.26–5.87, *p* = .011), neurologic disorders (adjusted OR 2.01, 95% CI: 1.01–3.99, *p* = .046), cardiogenic shock (adjusted OR 5.63, 95% CI: 2.76–11.48, *p* < .001), SBP <122 mmHg (adjusted OR 2.96, 95% CI: 1.46–6.02, *p* = .003) and WBC ≥11.3 × 10^9^/L (adjusted OR 2.06, 95% CI: 1.01–4.19, *p* = .047) were independent positive predictors, while chest pain was an independent negative predictor of in‐hospital death (adjusted OR .19, 95% CI: .06–.60, *p* = .005) (Table 3).

**TABLE 3:** Multivariate logistic regression analysis for in‐hospital death in patients with takotsubo syndrome in the training cohort.

	Unadjusted OR (95% CI)	*p*‐Value	Adjusted OR (95% CI)	*p*‐Value
Age ≥65 years	.71 (.42, 1.20)	.199	–	–
Male	1.77 (.98, 3.19)	.059	–	–
Race				
White	Reference		–	–
Black	.68 (.26, 1.79)	.436		
Asian	.89 (.39, 2.07)	.793		
Unknown	.99 (.29, 3.41)	.986		
BMI				
Normal weight/overweight	Reference		Reference	
Mild‐to‐moderate obesity	1.72 (.87, 3.43)	.120	2.18 (.90, 5.29)	.085
Severe obesity	5.65 (2.36, 13.54)	<.001	3.66 (1.13, 11.88)	.031
Underweight	2.90 (1.29, 6.56)	.010	3.57 (1.27, 10.05)	.016
Diabetes mellitus	1.64 (.93, 2.91)	.089	–	–
Atrial fibrillation	1.64 (.85, 3.17)	.144	–	–
Asthma	.23 (.03, 1.67)	.145	–	–
CKD	2.64 (1.46, 4.78)	.001	2.73 (1.26, 5.87)	.011
Neurologic disorders	1.52 (.90, 2.58)	.121	2.01 (1.01, 3.99)	.046
Malignancy	1.61 (.91, 2.84)	.104	–	–
Physical trigger	3.86 (1.72, 8.66)	.001	–	–
Chest pain	.12 (.04, .33)	<.001	.19 (.06, .60)	.005
Cardiogenic shock	6.90 (3.89, 12.24)	<.001	5.63 (2.76, 11.48)	<.001
Heart rate ≥95 bpm	1.43 (.84, 2.44)	.183	–	–
SBP <122 mmHg	3.60 (1.99, 6.53)	<.001	2.96 (1.46, 6.02)	.003
WBC ≥11.3 × 10^9^/L	2.58 (1.47, 4.55)	.001	2.06 (1.01, 4.19)	.047
ST‐segment elevation	.85 (.44, 1.64)	.622	–	–
ST‐segment depression	1.82 (.97, 3.42)	.062	–	–
QTc prolongation	.68 (.40, 1.17)	.167	–	–
LVEF ≤35%	2.19 (1.27, 3.79)	.005	–	–
RV involvement	1.79 (1.00, 3.21)	.049	–	–

Abbreviations: BMI, body mass index; CI, confidence interval; CKD, chronic kidney disease; LVEF, left ventricular ejection fraction; OR, odds ratio; RV, right ventricular; SBP, systolic blood pressure; WBC, white blood cell.

### 3.2 Nomogram construction and validation

**FIGURE 1:**
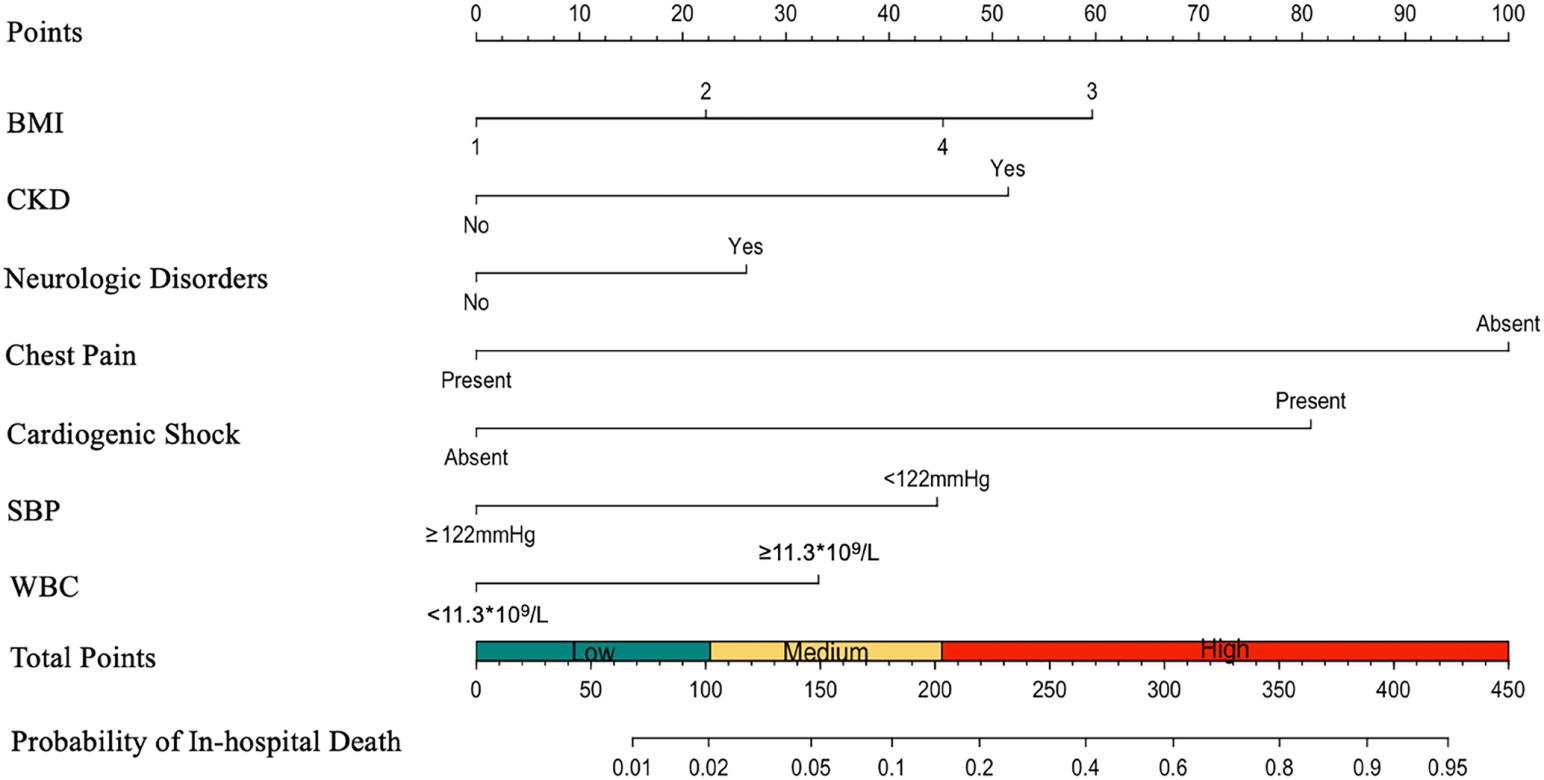
Nomogram for predicting in‐hospital death in patients with takotsubo syndrome. The total points were determined based on the sum of the points assigned to each variable in the nomogram. Patients were divided into three risk groups: Low‐risk (total points <102), medium‐risk (total points 102–203) and high‐risk (total points ≥203). For BMI, ‘1’ represented ‘normal weight/overweight’; ‘2’ represented ‘mild‐to‐moderate obesity’; ‘3’ represented ‘severe obesity’; ‘4’ represented ‘underweight’. BMI, body mass index; CKD, chronic kidney disease; SBP, systolic blood pressure; WBC, white blood cell.

A nomogram was constructed to predict in‐hospital death in TTS patients based on these seven independent variables (Figure 1). Total points were determined based on the sum of the points assigned to each variable in the nomogram, with higher total points corresponding to a higher risk of in‐hospital death. As an illustration, a TTS patient with mild obesity (22 points), history of CKD (51.5 points) and neurologic disorders (26 points), absence of chest pain (100 points), presence of cardiogenic shock (80.5 points), SBP 100 mmHg on admission (44.5 points) and WBC 16.5 × 10^9^/L on admission (33.5 points) would have a total of 358 points. This corresponds to a predicted probability of in‐hospital death of 82.1%.

**FIGURE 2:**
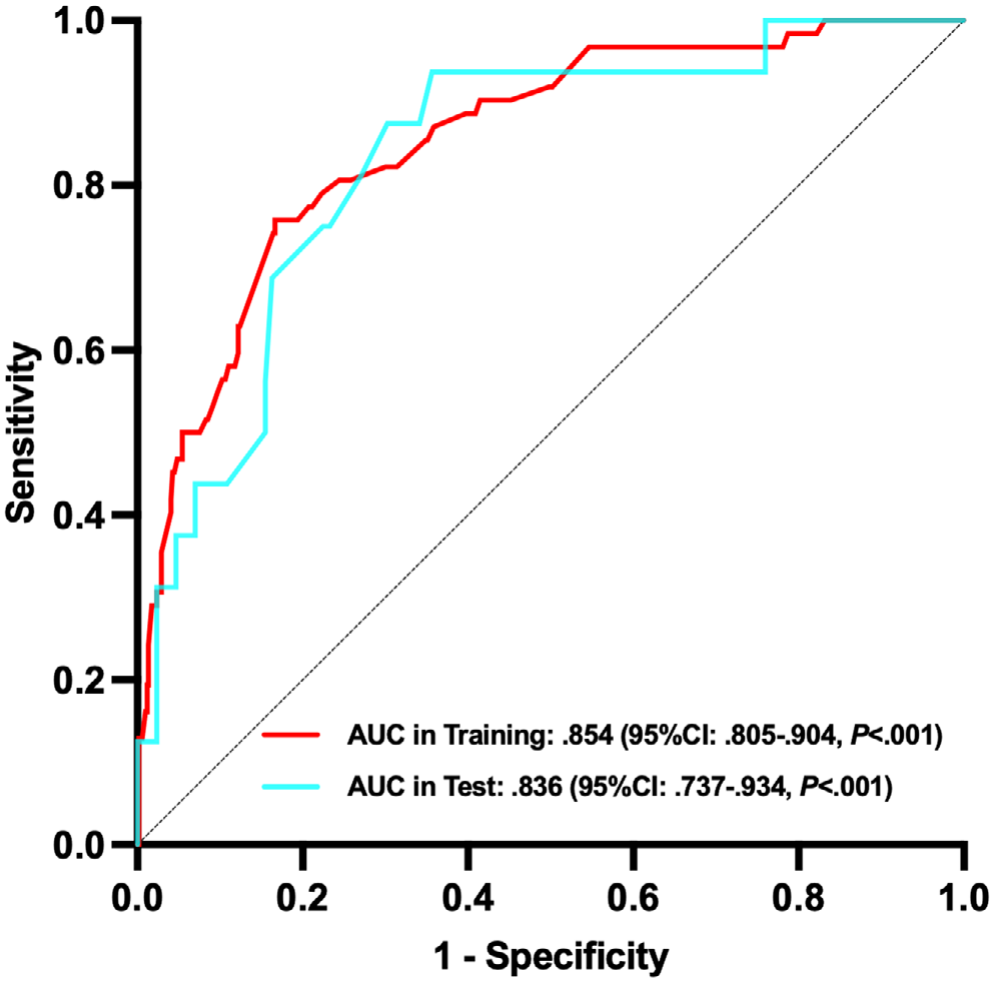
Receiver operating characteristic curves of the nomogram in the training and test cohorts. AUC, area under the curve; CI, confidence interval.

**FIGURE 3:**
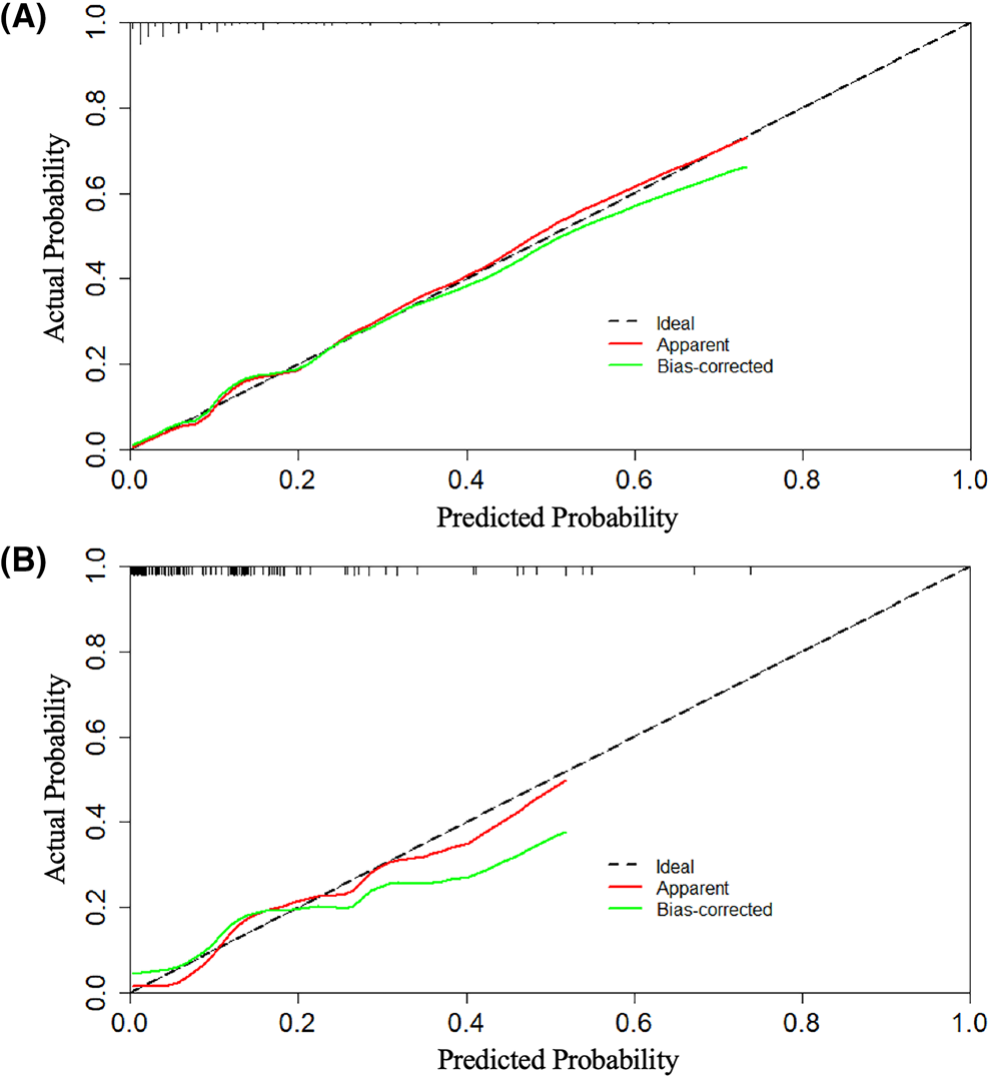
Calibration curves of the nomogram in the training (A) and test (B) cohorts. The red line represented the performance of the nomogram, the green line corrected the bias in the nomogram and the black dotted line represented the ideal reference line. The closer the red or green line is to the black dotted line, the more accurate the nomogram predicts the probability of in‐hospital death.

**FIGURE 4:**
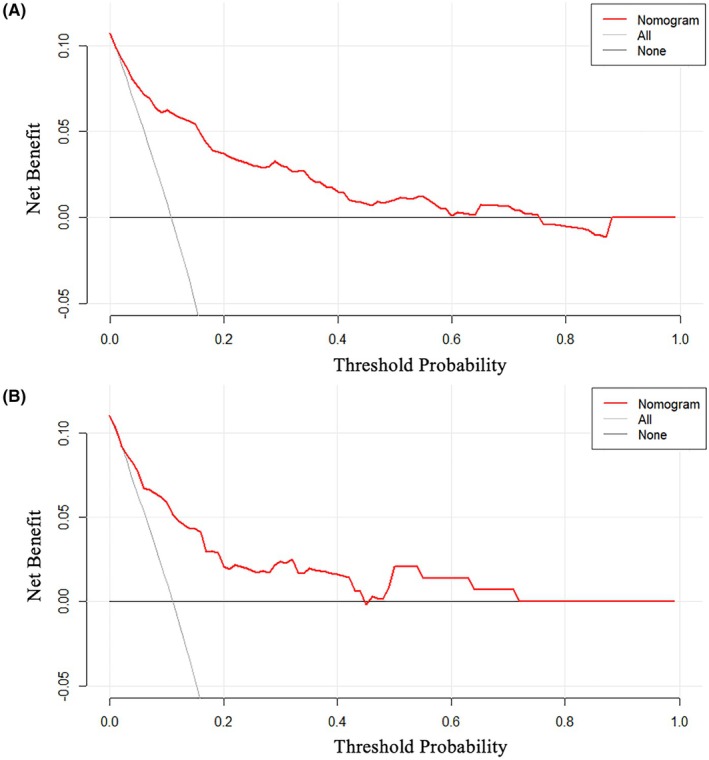
Decision curve analyses of the nomogram in the training (A) and test (B) cohorts. The grey line represented the net benefit of the strategy that all patients were treated. The black line represented the net benefit of the strategy that none of patients were treated. The red line represented the nomogram.

The nomogram demonstrated good predictive power, with AUCs of .854 (95% CI: .805–.904, *p* < .001) in the training cohort and .836 (95% CI: .737–.934, *p* < .001) in the test cohort (Figure 2). The calibration curves showed good consistency between the prediction of the nomogram and the actual observation in both the training and test cohorts (Hosmer‐Lemeshow *P*: .110 and .341, respectively) (Figure 3). Decision curve analyses indicated that the use of the nomogram to predict in‐hospital death in TTS patients could provide better net benefit compared to the ‘treat all’ or ‘treat none’ strategies when the threshold probability ranged from 2% to 75% in the training cohort and from 2% to 72% in the test cohort (Figure 4).

### 3.3 Risk stratification of the nomogram

**FIGURE 5:**
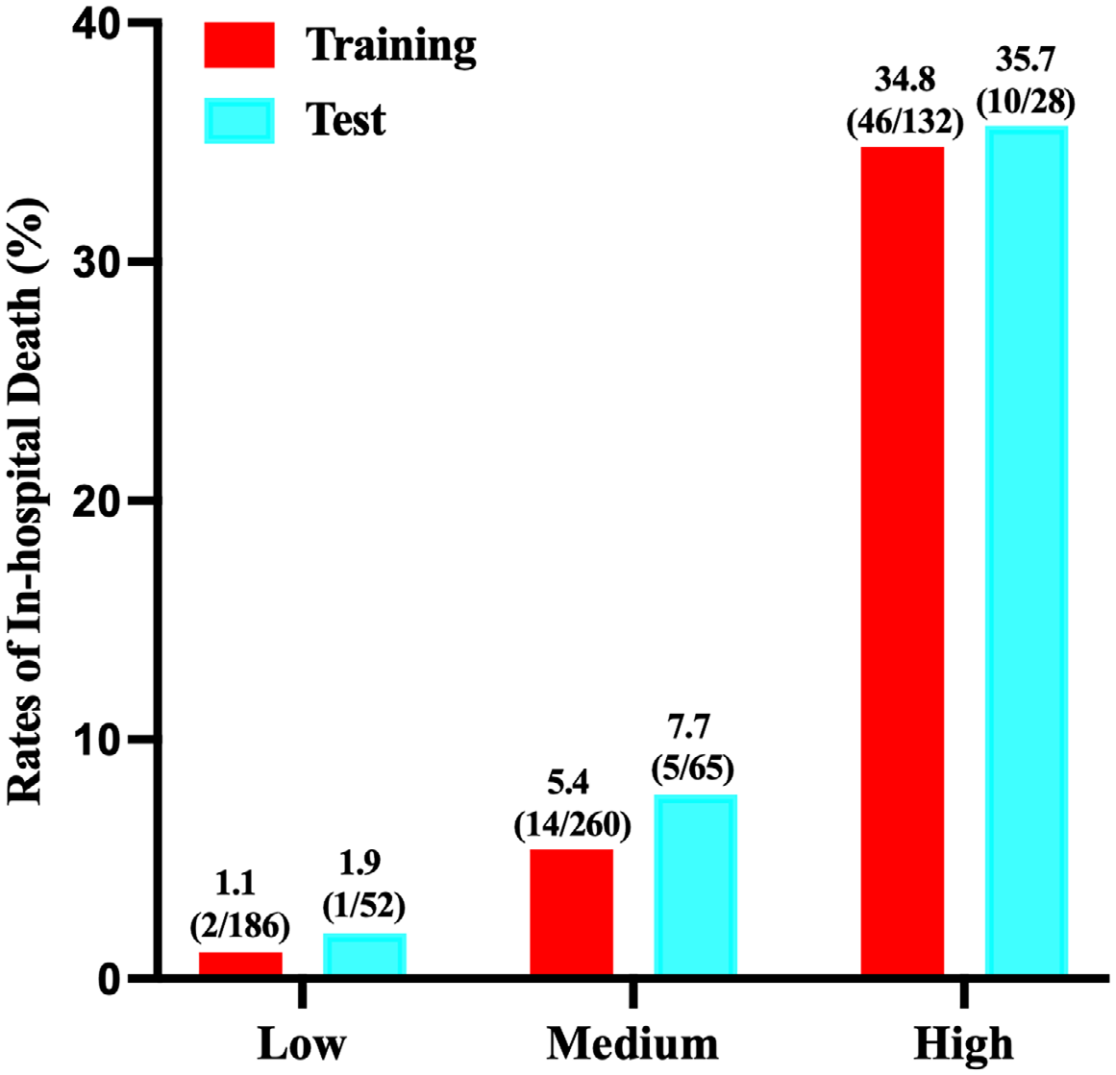
Rates of in‐hospital death in three different risk groups in the training and test cohorts.

Following the previous study,^15^ patients were stratified into three risk groups: low‐risk (total points <102), medium‐risk (total points 102–203) and high‐risk (total points ≥203). The risk of in‐hospital death significantly increased with the increase of the risk class (medium vs. low: OR 5.24, 95% CI: 1.18–23.32, *p* = .030; high vs. medium: OR 9.40, 95% CI: 4.92–17.94, *p* < .001). The rates of in‐hospital death in low‐, medium‐ and high‐risk were 1.1% (2/186), 5.4% (14/260) and 34.8% (46/132), respectively, in the training cohort, and 1.9% (1/52), 7.7% (5/65) and 35.7% (10/28), respectively, in the test cohort (Figure 5).

### 3.4 Predictive value of the nomogram compared with other prognostic scores for in‐hospital death in TTS patients in the training and test cohorts

Compared to the InterTAK Prognostic Score,^9^ the nomogram improved the prognostic performance for predicting in‐hospital death in TTS patients, but the improvement was not statistically significant in the test cohort (C‐index: .854 vs. .753, *p* = .002 [Training]; .836 vs. .778, *p* = .530 [Test]). It also improved the NRI (.413, *p* < .001 [Training]; .248, *p* = .187 [Test]) and IDI (.156, *p* < .001 [Training]; .039, *p* = .647 [Test]) for the prognostic value of in‐hospital death, but these improvements were not statistically significant in the test cohort.

Compared to the GEIST Prognostic Score,^10^ the nomogram improved the prognostic performance for predicting in‐hospital death in TTS patients, but the improvement did not reach statistical significance in the test cohort (C‐index: .854 vs. .655, *p* < .001 [Training]; .836 vs. .744, *p* = .334 [Test]). It also improved the IDI for the prognostic value of in‐hospital death, but it did not reach statistical significance in the test cohort (.211, *p* < .001 [Training]; .113, *p* = .061 [Test]). Importantly, it significantly improved the NRI for the prognostic value of in‐hospital death in both cohorts (.786, *p* < .001 [Training]; .466, *p* = .017 [Test]) (Table 4).

**TABLE 4:** Comparison of predictive value for in‐hospital death between the nomogram and other prognostic scores in patients with takotsubo syndrome in the training and test cohorts.

Models	C‐index (95% CI)	*p*‐Value	NRI (95% CI)	*p*‐Value	IDI (95% CI)	*p*‐Value
Training						
Nomogram^a^	.854 (.805, .904)	Reference	Reference		Reference	
InterTAK Prognostic Score^b^	.753 (.697, .809)	.002	−.413 (−.576, −.249)	<.001	−.156 (−.216, −.096)	<.001
GEIST Prognostic Score^c^	.655 (.584, .726)	<.001	−.786 (−.967, −.605)	<.001	−.211 (−.272, −.150)	<.001
Test						
Nomogram^a^	.836 (.737, .934)	Reference	Reference		Reference	
InterTAK Prognostic Score^b^	.778 (.649, .906)	.530	−.248 (−.617, .120)	.187	−.039 (−.203, .126)	.647
GEIST Prognostic Score^c^	.744 (.608, .881)	.334	−.466 (−.849, −.082)	.017	−.113 (−.230, .005)	.061

Abbreviations: BMI, body mass index; CI, confidence interval; CKD, chronic kidney disease; IDI, integrated discrimination improvement; LVEF, left ventricular ejection fraction; NRI, net reclassification improvement; RV, right ventricular; SBP, systolic blood pressure; TTS, takotsubo syndrome; WBC, white blood cell.

^a^ Includes BMI, CKD, neurologic disorders, chest pain, cardiogenic shock, SBP <122 mmHg, WBC ≥11.3 × 10^9^/L. A total of 122 TTS patients from the InterTAK registry were enrolled in this study, and 47 TTS patients overlapped with those in the InterTAK Prognostic Score.

^b^ Includes TTS secondary to neurologic disorders, TTS secondary to physical activities, medical conditions or procedures, TTS without an identifiable triggering factor, age >70 years, SBP <119 mmHg on admission, diabetes mellitus, LVEF ≤45% on admission, male sex, heart rate >94 bpm on admission.

^c^ Includes male sex, history of neurologic disorders, RV involvement, LVEF.

### 3.5 External validation

In‐hospital death was 6.6% in the external validation cohort. The nomogram demonstrated good predictive power, with AUC of .838 (95% CI: .663–1.000, *p* = .003) in the external validation cohort (Figure S2). The calibration curves showed good consistency between the prediction of the nomogram and the actual observation in the external validation cohort (Hosmer‐Lemeshow *P*: .233) (Figure S3).

## 4 DISCUSSION

The main findings of our study are as follows: (1) BMI, CKD, neurologic disorders, cardiogenic shock, low SBP and abnormal WBC were independent positive predictors, while chest pain was an independent negative predictor of in‐hospital death in a multinational cohort of TTS patients. (2) A new nomogram was developed for predicting in‐hospital death in TTS patients based on these seven independent variables and showed good discriminative and calibration abilities. (3) The risk of in‐hospital death significantly increased with the increase of established risk class in a multinational cohort of TTS patients.

Consistent with previous findings,^10^, ^17^, ^18^, ^19^, ^20^ our study showed that CKD and neurologic disorders significantly increased the risk of in‐hospital death in a multinational cohort of TTS patients. Although our study also confirmed previous findings^15^, ^21^, ^22^, ^23^ that presence of cardiogenic shock on admission significantly increased the risk of in‐hospital death, SBP itself became an independent predictor of in‐hospital death in TTS patients. Particularly, the risk of in‐hospital death increased with the decrease of SBP levels when SBP was <122 mmHg. Therefore, low SBP in TTS patients on admission raises a red flag and warrants close clinical monitoring and timely interventions. Consistent with previous studies,^15^, ^24^ WBC was an independent predictor of in‐hospital death in our study as well. Especially, the risk of in‐hospital death increased with the increase of WBC counts when WBC was ≥11.3 × 10^9^/L. The activation of systemic inflammatory response may become a potential clinical marker for adverse outcomes in TTS patients.^25^, ^26^

The correlation between BMI and outcomes in TTS patients is interesting. A previous Japanese retrospective study showed that underweight was significantly associated with higher in‐hospital mortality compared with normal weight (18.5–22.9 kg/m^2^), while no significant association was noted between obesity (≥27.5 kg/m^2^) and normal weight.^27^ Meanwhile, other studies also suggested that low BMI was associated with in‐hospital death,^4^ and the rate of in‐hospital death was similar between obesity and nonobesity.^28^ Of note, in our study, although underweight still posed a higher risk of in‐hospital death, severe obesity was significantly associated with higher in‐hospital death compared to normal weight/overweight, which may be associated with more cardiogenic shock events (31.3% vs. 12.5%, *p* = .006) and WBC ≥11.3 × 10^9^/L (68.8% vs. 49.1%, *p* = .031) (Table S4). In our study dataset, 96% of patients with moderate or severe obesity were from the USA patients (AmSC Research Network data), which may become an essential contributor to help explore the factual relationship between BMI and outcomes in TTS patients.

Of note, our study showed that TTS patients with chest pain had a lower risk of in‐hospital death. Compared to patients without chest pain, those with chest pain were more frequently female and less likely to have atrial fibrillation, malignancy, neurologic disorders, physical trigger, SBP <122 mmHg and WBC ≥11.3 × 10^9^/L. Additionally, they had lower heart rate and better LV and RV function in echocardiograms (Table S5). Previous studies showed that the patients who were chest pain‐free had higher in‐hospital death during acute myocardial infarction or acute pulmonary embolism, which was attributed to worse baseline characteristics, delayed presentation to the hospital and decreased likelihood to receive standard therapies.^29^, ^30^, ^31^, ^32^ As one of the most common TTS associated symptoms,^1^ chest pain appears to be associated with a lower risk of in‐hospital complications/death in TTS patients as well. The underlying pathophysiology and clinical significance likely become important research targets down the road.

In addition, the clinical and laboratory features in this nomogram can be obtained rapidly and conveniently during routine clinical practice, making it clinically practical and broadly applicable. This may provide a streamlined and interpretable tool tailored for bedside use, highlighting individualized risk assessment in hospitalized TTS patients and supporting personalized treatment strategies to reduce adverse events.

There are several limitations in our study. First of all, we only requested and obtained the clinical data of TTS patients whose raw echocardiographic imaging data were available from two international/national registries as the training/test cohorts, and those without raw echocardiographic imaging data from the ChiTTS registry as the external validation cohort, which may lead to a relatively small study size and selection bias in the present clinical study. Secondly, the rate of in‐hospital death was 10.3% in our study, which appeared to be higher than previous investigations.^4^, ^10^, ^15^, ^16^, ^33^ Since most TTS patients were from two major tertiary medical centers in the USA (Washington University in St. Louis and University of Iowa), those patients may have more severe clinical manifestations and physical triggers compared to other TTS patients from regional and rural hospitals. Thirdly, data in this study spanned from 2004 to 2024 (Figure S4), and there is possible heterogeneity across the three registries/research network, especially regarding inclusion criteria, case mix, and data completeness. Finally, as a retrospective study, the established nomogram needs to be tested with large‐scale prospective trials, and we are collaborating with more international TTS registries for this research purpose.

## 5 CONCLUSIONS

The nomogram, composed of BMI, CKD, neurologic disorders, chest pain, cardiogenic shock, low SBP and abnormal WBC, helps predict in‐hospital death in TTS patients.

## AUTHOR CONTRIBUTIONS

Study design: YC, AC, FC, XW, JC and KL; Data collection: YC, AC, FC, DDV, MW, PT, TKC and JC; Data analysis: YC; Writing‐original draft: YC and AC; Writing‐review and editing: YC, AC, FC, DDV, CT, XW, JC and KL; Project administration and supervision: XW, JC and KL. All authors have revised and approved the submitted manuscript.

## FUNDING INFORMATION

This work was supported by National Institutes of Health Award to KL and XW (R01HL171624‐01).

## CONFLICT OF INTEREST STATEMENT

All authors have no competing interests to declare.

## Supporting information


Appendix S1.

## Data Availability

The data underlying this study will be available upon reasonable request to the corresponding author.

## References

[1] Ghadri JR , Wittstein IS , Prasad A , et al. International expert consensus document on Takotsubo syndrome (part I): clinical characteristics, diagnostic criteria, and pathophysiology. Eur Heart J. 2018;39:2032‐2046. doi:10.1093/eurheartj/ehy076 29850871 PMC5991216

[2] Citro R , Radano I , Bellino M , et al. Epidemiology, pathogenesis, and clinical course of Takotsubo syndrome. Heart Fail Clin. 2022;18:125‐137. doi:10.1016/j.hfc.2021.08.001 34776074

[3] Singh T , Khan H , Gamble DT , Scally C , Newby DE , Dawson D . Takotsubo syndrome: pathophysiology, emerging concepts, and clinical implications. Circulation. 2022;145:1002‐1019. doi:10.1161/CIRCULATIONAHA.121.055854 35344411 PMC7612566

[4] Arao K , Yoshikawa T , Isogai T , et al. A study of takotsubo syndrome over 9 years at the Tokyo cardiovascular care unit network registry. J Cardiol. 2023;82:93‐99. doi:10.1016/j.jjcc.2022.12.011 36640906

[5] Ghadri JR , Wittstein IS , Prasad A , et al. International expert consensus document on Takotsubo syndrome (part II): diagnostic workup, outcome, and management. Eur Heart J. 2018;39:2047‐2062. doi:10.1093/eurheartj/ehy077 29850820 PMC5991205

[6] Templin C , Ghadri JR , Diekmann J , et al. Clinical features and outcomes of Takotsubo (stress) cardiomyopathy. N Engl J Med. 2015;373:929‐938. doi:10.1056/NEJMoa1406761 26332547

[7] Ghadri JR , Kato K , Cammann VL , et al. Long‐term prognosis of patients with Takotsubo syndrome. J Am Coll Cardiol. 2018;72:874‐882. doi:10.1016/j.jacc.2018.06.016 30115226

[8] Schweiger V , Cammann VL , Crisci G , et al. Temporal trends in Takotsubo syndrome: Results from the international Takotsubo registry. J Am Coll Cardiol. 2024;84:1178‐1189. doi:10.1016/j.jacc.2024.05.076 39217551

[9] Wischnewsky MB , Candreva A , Bacchi B , et al. Prediction of short‐ and long‐term mortality in takotsubo syndrome: the InterTAK prognostic score. Eur J Heart Fail. 2019;21:1469‐1472. doi:10.1002/ejhf.1561 31452320

[10] Santoro F , Nunez Gil IJ , Stiermaier T , et al. Assessment of the German and Italian stress cardiomyopathy score for risk stratification for in‐hospital complications in patients with Takotsubo syndrome. JAMA Cardiol. 2019;4:892‐899. doi:10.1001/jamacardio.2019.2597 31389988 PMC6686773

[11] Ghadri JR , Cammann VL , Templin C . The international Takotsubo registry: rationale, design, objectives, and first results. Heart Fail Clin. 2016;12:597‐603. doi:10.1016/j.hfc.2016.06.010 27638029

[12] Chong TK , Chen J , Lyu L , et al. Clinical characteristics and outcome correlates of Chinese patients with takotsubo syndrome: Results from the first Chinese takotsubo syndrome registry. Int J Cardiol. 2023;387:131129. doi:10.1016/j.ijcard.2023.131129 37355242

[13] Zaman F , Ponnapureddy R , Wang YG , et al. Spatio‐temporal hybrid neural networks reduce erroneous human “judgement calls” in the diagnosis of Takotsubo syndrome. EClinicalMedicine. 2021;40:101115. doi:10.1016/j.eclinm.2021.101115 34522872 PMC8426197

[14] Zaman F , Isom N , Chang A , et al. Deep learning from atrioventricular plane displacement in patients with Takotsubo syndrome: lighting up the black‐box. Eur Heart J Digit Health. 2024;5:134‐143. doi:10.1093/ehjdh/ztad077 38505490 PMC10944681

[15] De Filippo O , Cammann VL , Pancotti C , et al. Machine learning‐based prediction of in‐hospital death for patients with takotsubo syndrome: the InterTAK‐ML model. Eur J Heart Fail. 2023;25:2299‐2311. doi:10.1002/ejhf.2983 37522520

[16] Agrawal A , Bhagat U , Arockiam AD , et al. Machine learning risk‐prediction model for in‐hospital mortality in Takotsubo cardiomyopathy. Int J Cardiol. 2025;430:133181. doi:10.1016/j.ijcard.2025.133181 40120825

[17] Lu X , Li P , Teng C , et al. Prognostic factors of Takotsubo cardiomyopathy: a systematic review. ESC Heart Fail. 2021;8:3663‐3689. doi:10.1002/ehf2.13531 34374223 PMC8497208

[18] Ando K , Sukekawa H , Takahata A , et al. Renal dysfunction indicative of outcomes in hospitalized patients with takotsubo syndrome. Eur Heart J Acute Cardiovasc Care. 2018;7:723‐731. doi:10.1177/2048872617715019 28593801

[19] Ogunniyi KE , Akinmoju OD , Olatunji G , et al. When is the broken heart most dangerous? Assessing risk factors to predict inpatient death in Takotsubo cardiomyopathy: analysis of the National Inpatient Sample for 2021. J Am Heart Assoc. 2025;14:e040167. doi:10.1161/JAHA.124.040167 40673530 PMC12449941

[20] Santoro F , Nunez Gil IJ , Arcari L , et al. Neurological disorders in Takotsubo syndrome: clinical phenotypes and outcomes. J Am Heart Assoc. 2024;13:e032128. doi:10.1161/JAHA.123.032128 38353238 PMC11010100

[21] Almendro‐Delia M , Nunez‐Gil IJ , Lobo M , et al. Short‐ and long‐term prognostic relevance of cardiogenic shock in Takotsubo syndrome: Results from the RETAKO registry. JACC Heart Fail. 2018;6:928‐936. doi:10.1016/j.jchf.2018.05.015 30316938

[22] Stiermaier T , Eitel C , Desch S , et al. Incidence, determinants and prognostic relevance of cardiogenic shock in patients with Takotsubo cardiomyopathy. Eur Heart J Acute Cardiovasc Care. 2016;5:489‐496. doi:10.1177/2048872615612456 26474843

[23] Di Vece D , Citro R , Cammann VL , et al. Outcomes associated with cardiogenic shock in Takotsubo syndrome. Circulation. 2019;139:413‐415. doi:10.1161/CIRCULATIONAHA.118.036164 30586690

[24] Murakami T , Yoshikawa T , Maekawa Y , et al. Characterization of predictors of in‐hospital cardiac complications of takotsubo cardiomyopathy: multi‐center registry from Tokyo CCU network. J Cardiol. 2014;63:269‐273. doi:10.1016/j.jjcc.2013.09.003 24139869

[25] Scally C , Abbas H , Ahearn T , et al. Myocardial and systemic inflammation in acute stress‐induced (Takotsubo) cardiomyopathy. Circulation. 2019;139:1581‐1592. doi:10.1161/CIRCULATIONAHA.118.037975 30586731 PMC6438459

[26] Dusi V , Ghidoni A , Ravera A , De Ferrari GM , Calvillo L . Chemokines and heart disease: a network connecting cardiovascular biology to immune and autonomic nervous systems. Mediat Inflamm. 2016;2016:5902947. doi:10.1155/2016/5902947 PMC4868905 27242392

[27] Isogai T , Okada A , Morita K , et al. Body mass index and outcomes in patients with Takotsubo syndrome: A Nationwide retrospective cohort study. Cardiology. 2024;149:314‐324. doi:10.1159/000537971 38387447 PMC11309069

[28] Desai R , Singh S , Baikpour M , et al. Does obesity affect the outcomes in takotsubo cardiomyopathy? Analysis of the Nationwide inpatient sample database, 2010–2014. Clin Cardiol. 2018;41:1028‐1034. doi:10.1002/clc.22999 29917260 PMC6489855

[29] Chien DK , Lee SY , Hung CL , Sun FJ , Lin MR , Chang WH . Do patients with non‐ST‐elevation myocardial infarction without chest pain suffer a poor prognosis? Taiwan J Obstet Gynecol. 2019;58:788‐792. doi:10.1016/j.tjog.2019.09.010 31759528

[30] Song CX , Fu R , Yang JG , et al. Angiographic characteristics and in‐hospital mortality among patients with ST‐segment elevation myocardial infarction presenting without typical chest pain: an analysis of China acute myocardial infarction registry. Chin Med J. 2019;132:2286‐2291. doi:10.1097/CM9.0000000000000432 31567475 PMC6819048

[31] Dorsch MF , Lawrance RA , Sapsford RJ , et al. Poor prognosis of patients presenting with symptomatic myocardial infarction but without chest pain. Heart. 2001;86:494‐498. doi:10.1136/heart.86.5.494 11602537 PMC1729984

[32] Wong CC , Ng AC , Lau JK , et al. The prognostic impact of chest pain in 1306 patients presenting with confirmed acute pulmonary embolism. Int J Cardiol. 2016;221:794‐799. doi:10.1016/j.ijcard.2016.07.129 27428323

[33] Akashi YJ , Goldstein DS , Barbaro G , Ueyama T . Takotsubo cardiomyopathy: A new form of acute, reversible heart failure. Circulation. 2008;118:2754‐2762. doi:10.1161/CIRCULATIONAHA.108.767012 19106400 PMC4893309

