# Supplementary material for: A nomogram for predicting in‐hospital death in a multinational cohort of patients with takotsubo syndrome
Source: Eur J Clin Invest. 2026 Mar 26;56(4):e70190. doi: 10.1111/eci.70190 (PMC13022065; doi:10.1111/eci.70190)
Supplement: Supplementary file 1 — Appendix S1. [file ECI-56-e70190-s001.zip › eci70190-sup-0005-TableS1-S5-FigureS1-S4@Supplementary_Materials.docx]

**TABLE S1 Results of missing variables in the training/test cohorts**

|  | Missing rate (%) |
| --- | --- |
| Age | 0 |
| Sex | 0 |
| Race | 0 |
| BMI | 5.1 |
| Current smoker | 1.4 |
| Hypertension | .1 |
| Diabetes mellitus | 0 |
| Hyperlipidemia | .3 |
| Atrial fibrillation | 1.0 |
| Coronary artery disease | 3.0 |
| COPD | .3 |
| Asthma | .3 |
| CKD | 1.9 |
| Malignancy | .3 |
| Psychiatric disorders | .1 |
| Neurologic disorders  Acute neurologic disorders | .1  .8 |
| Physical trigger | 0 |
| Chest pain | 2.4 |
| Shortness of breath | 2.1 |
| Cardiogenic shock | 0 |
| Heart rate | 2.6 |
| SBP | 2.8 |
| DBP | 2.8 |
| Troponin | 3.6 |
| WBC | 1.8 |
| Creatinine | 6.9 |
| ST-segment elevation | 5.0 |
| ST-segment depression | 5.5 |
| T-wave inversion | 5.8 |
| QTc prolongation | 7.3 |
| Type of TTS | 0 |
| LVEF | .1 |
| RV involvement | 3.5 |

Abbreviations: BMI, body mass index; COPD, chronic obstructive pulmonary disease; CKD, chronic kidney disease; SBP, systolic blood pressure; DBP, diastolic blood pressure; WBC, white blood cell; TTS, takotsubo syndrome; LVEF, left ventricular ejection fraction; RV, right ventricular.

**TABLE S2 Results of missing variables in the external validation cohort**

|  | Missing rate (%) |
| --- | --- |
| BMI | 0 |
| CKD | 0 |
| Neurologic disorders | 0 |
| Chest pain | 0 |
| Cardiogenic shock | 0 |
| SBP | 0 |
| WBC | 5.7 |

Abbreviations: BMI, body mass index; CKD, chronic kidney disease; SBP, systolic blood pressure; WBC, white blood cell.

**TABLE S3 Univariate logistic regression analysis between BMI and in-hospital death in patients with takotsubo syndrome in the training cohort**

|  | OR (95%CI) | *P*-value |
| --- | --- | --- |
| Normal weight | reference |  |
| Overweight | .69 (.32, 1.48) | .343 |
| Mild obesity | 1.55 (.67, 3.57) | .309 |
| Moderate obesity | 1.37 (.44, 4.29) | .585 |
| Severe obesity | 4.88 (1.95, 12.20) | .001 |
| Underweight | 2.51 (1.06, 5.93) | .037 |

Abbreviations: BMI, body mass index; OR, odds ratio; CI, confidence interval.

**TABLE S4 Baseline clinical features between obesity and normal weight/overweight in the training cohort**

|  | Normal weight/overweight (n=497) | Mild-to-moderate obesity (n=133) | Severe obesity (n=32) | *P*-value^#^ | *P*-value^*^ |  |
| --- | --- | --- | --- | --- | --- | --- |
| Age, years | 64.8±15.3 | 64.2±13.4 | 58.2±14.3 | .670 | .018 |  |
| Male, n (%) | 110 (22.1) | 22 (16.5) | 4 (12.5) | .159 | .199 |  |
| Race, n (%) |  |  |  | <.001 | .064 |  |
| White  Black  Asian  Unknown | 359 (72.2)  44 (8.9)  70 (14.1)  24 (4.8) | 116 (87.2)  15 (11.3)  0  2 (1.5) | 24 (75.0)  5 (15.6)  0  3 (9.4) |  |  |  |
| Hypertension, n (%) | 291 (58.6) | 91 (68.4) | 23 (71.9) | .039 | .137 |  |
| Diabetes mellitus, n (%) | 96 (19.3) | 50 (37.6) | 14 (43.8) | <.001 | .001 |  |
| Hyperlipidemia, n (%) | 219 (44.1) | 74 (55.6) | 21 (65.6) | .017 | .018 |  |
| Atrial fibrillation, n (%) | 71 (14.3) | 22 (16.5) | 6 (18.8) | .515 | .663 |  |
| Coronary artery disease, n (%) | 99 (19.9) | 27 (20.3) | 8 (25.0) | .922 | .488 |  |
| COPD, n (%) | 56 (11.3) | 24 (18.0) | 8 (25.0) | .037 | .042 |  |
| Asthma, n (%) | 30 (6.0) | 12 (9.0) | 3 (9.4) | .220 | .704 |  |
| CKD, n (%) | 74 (14.9) | 25 (18.8) | 8 (25.0) | .271 | .126 |  |
| Malignancy, n (%) | 133 (26.8) | 30 (22.6) | 6 (18.8) | .325 | .318 |  |
| Psychiatric disorders, n (%) | 169 (34.0) | 63 (47.4) | 16 (50.0) | .005 | .066 |  |
| Neurologic disorders, n (%)  Acute neurologic disorders, n (%) | 182 (36.6)  94 (18.9) | 57 (42.9)  26 (19.5) | 12 (37.5)  4 (12.5) | .188  .868 | .920  .365 |  |
| Physical trigger, n (%) | 339 (68.2) | 91 (68.4) | 27 (84.4) | .963 | .055 |  |
| Chest pain, n (%) | 182 (36.6) | 47 (35.3) | 10 (31.3) | .785 | .540 |  |
| Shortness of breath, n (%) | 231 (46.5) | 67 (50.4) | 21 (65.6) | .424 | .036 |  |
| Cardiogenic shock, n (%) | 62 (12.5) | 17 (12.8) | 10 (31.3) | .924 | .006 |  |
| Heart rate, bpm | 95.4±22.7 | 91.8±21.2 | 101.1±23.3 | .101 | .173 |  |
| SBP <122 mmHg, n (%) | 245 (49.3) | 58 (43.6) | 17 (53.1) | .244 | .675 |  |
| DBP, mmHg | 75.5±16.8 | 74.2±16.4 | 75.4±22.0 | .431 | .980 |  |
| Troponin, ng/ml | .54 (.11, 2.64) | .37 (.08, 1.13) | .97 (.11, 1.95) | .020 | .898 |  |
| WBC ≥11.3*10**^9^**/L, n (%) | 244 (49.1) | 63 (47.4) | 22 (68.8) | .724 | .031 |  |
| Creatinine, mg/dl | .9 (.7, 1.2) | 1.0 (.7, 1.3) | 1.1 (.8, 1.6) | .079 | .020 |  |
| ST-segment elevation, n (%) | 116 (23.3) | 18 (13.5) | 8 (25.0) | .014 | .830 |  |
| ST-segment depression, n (%) | 86 (17.3) | 19 (14.3) | 4 (12.5) | .407 | .483 |  |
| T-wave inversion, n (%) | 249 (50.1) | 81 (60.9) | 16 (50.0) | .027 | .991 |  |
| QTc prolongation, n (%) | 319 (64.2) | 93 (69.9) | 16 (50.0) | .216 | .107 |  |
| Type of TTS, n (%) |  |  |  | .101 | .148 |  |
| Apical  Mid-ventricular  Basal  Focal  Global | 357 (71.8)  45 (9.1)  34 (6.8)  28 (5.6)  33 (6.6) | 98 (73.7)  8 (6.0)  6 (4.5)  15 (11.3)  6 (4.5) | 28 (87.5)  0  0  1 (3.1)  3 (9.4) |  |  |  |
| LVEF, % | 38.2±12.5 | 38.3±11.8 | 36.7±9.3 | .933 | .366 |  |
| RV involvement, n (%) | 101 (20.3) | 19 (14.3) | 10 (31.3) | .115 | .141 |  |

Abbreviations: COPD, chronic obstructive pulmonary disease; CKD, chronic kidney disease; SBP, systolic blood pressure; DBP, diastolic blood pressure; WBC, white blood cell; TTS, takotsubo syndrome; LVEF, left ventricular ejection fraction; RV, right ventricular.

^#^: mild-to-moderate obesity vs. normal weight/overweight

*: severe obesity vs. normal weight/overweight

**TABLE S5 Baseline clinical features of patients with and without chest pain in the training cohort**

|  | Chest pain (n=196) | No chest pain (n=382) | *P*-value |  |
| --- | --- | --- | --- | --- |
| Age, years | 65.1±13.3 | 63.7±16.3 | .288 |  |
| Male, n (%) | 27 (13.8) | 88 (23.0) | .008 |  |
| Race, n (%) |  |  | .900 |  |
| White  Black  Asian  Unknown | 141 (71.9)  20 (10.2)  26 (13.3)  9 (4.6) | 278 (72.8)  43 (11.3)  43 (11.3)  18 (4.7) |  |  |
| BMI, n (%) |  |  | .852 |  |
| Underweight | 13 (6.6) | 33 (8.6) |  |  |
| Normal weight/overweight | 138 (70.4) | 263 (68.8) |  |  |
| Mild-to-moderate obesity | 36 (18.4) | 67 (17.5) |  |  |
| Severe obesity | 9 (4.6) | 19 (5.0) |  |  |
| Hypertension, n (%) | 128 (65.3) | 225 (58.9) | .135 |  |
| Diabetes mellitus, n (%) | 39 (19.9) | 97 (25.4) | .140 |  |
| Hyperlipidemia, n (%) | 97 (49.5) | 170 (44.5) | .255 |  |
| Atrial fibrillation, n (%) | 16 (8.2) | 69 (18.1) | .001 |  |
| Coronary artery disease, n (%) | 48 (24.5) | 67 (17.5) | .048 |  |
| COPD, n (%) | 30 (15.3) | 56 (14.7) | .836 |  |
| Asthma, n (%) | 16 (8.2) | 20 (5.2) | .168 |  |
| CKD, n (%) | 30 (15.3) | 63 (16.5) | .713 |  |
| Malignancy, n (%) | 34 (17.3) | 104 (27.2) | .008 |  |
| Psychiatric disorders, n (%) | 76 (38.8) | 143 (37.4) | .753 |  |
| Neurologic disorders, n (%)  Acute neurologic disorders, n (%) | 55 (28.1)  10 (5.1) | 163 (42.7)  97 (25.4) | .001  <.001 |  |
| Physical trigger, n (%) | 86 (43.9) | 315 (82.5) | <.001 |  |
| Shortness of breath, n (%) | 109 (55.6) | 170 (44.5) | .011 |  |
| Cardiogenic shock, n (%) | 22 (11.2) | 61 (16.0) | .124 |  |
| Heart rate, bpm | 91.5±22.8 | 98.0±23.1 | .001 |  |
| SBP <122 mmHg, n (%) | 78 (39.8) | 197 (51.6) | .007 |  |
| DBP, mmHg | 76.3±16.3 | 75.0±18.2 | .396 |  |
| Troponin, ng/ml | .92 (.22, 6.17) | .38 (.07, 1.20) | <.001 |  |
| WBC ≥11.3*10**^9^**/L, n (%) | 84 (42.9) | 200 (52.4) | .031 |  |
| Creatinine, mg/dl | .9 (.7, 1.1) | .9 (.7, 1.3) | .008 |  |
| ST-segment elevation, n (%) | 53 (27.0) | 73 (19.1) | .029 |  |
| ST-segment depression, n (%) | 18 (9.2) | 74 (19.4) | .002 |  |
| T-wave inversion, n (%) | 114 (58.2) | 194 (50.8) | .092 |  |
| QTc prolongation, n (%) | 125 (63.8) | 265 (69.4) | .174 |  |
| Type of TTS, n (%) |  |  | .010 |  |
| Apical  Mid-ventricular  Basal  Focal  Global | 139 (70.9)  22 (11.2)  9 (4.6)  18 (9.2)  8 (4.1) | 280 (73.3)  21 (5.5)  28 (7.3)  21 (5.5)  32 (8.4) |  |  |
| LVEF, % | 40.0±12.7 | 36.9±11.5 | .003 |  |
| RV involvement, n (%) | 31 (15.8) | 90 (23.6) | .030 |  |

Abbreviations: BMI, body mass index; COPD, chronic obstructive pulmonary disease; CKD, chronic kidney disease; SBP, systolic blood pressure; DBP, diastolic blood pressure; WBC, white blood cell; TTS, takotsubo syndrome; LVEF, left ventricular ejection fraction; RV, right ventricular.

**Supplementary Figure Legend:**

**FIGURE S1 Association between variables and in-hospital death using restricted cubic spline curve. (A) Association between BMI and in-hospital death. (B) Association between heart rate and in-hospital death. (C) Association between SBP and in-hospital death. (D) Association between WBC and in-hospital death. BMI, body mass index; SBP, systolic blood pressure; WBC, white blood cell; OR, odds ratio; CI, confidence interval.**

**FIGURE S2 Receiver operating characteristic curve of the nomogram in the external validation cohort. AUC, area under the curve; CI, confidence interval.**

**FIGURE S3 Calibration curves of the nomogram in the external validation cohort. The red line represented the performance of the nomogram, the green line corrected the bias in the nomogram, and the black dotted line represented the ideal reference line. The closer the red or green line is to the black dotted line, the more accurate the nomogram predicts the probability of in-hospital death.**

**FIGURE S4 Numbers of TTS patients and in-hospital death in the training/test cohorts from 2004 to 2024. TTS, takotsubo syndrome.**
