# Supplementary figures and images for: A nomogram for predicting in‐hospital death in a multinational cohort of patients with takotsubo syndrome
Source: Eur J Clin Invest. 2026 Mar 26;56(4):e70190. doi: 10.1111/eci.70190 (PMC13022065; doi:10.1111/eci.70190)

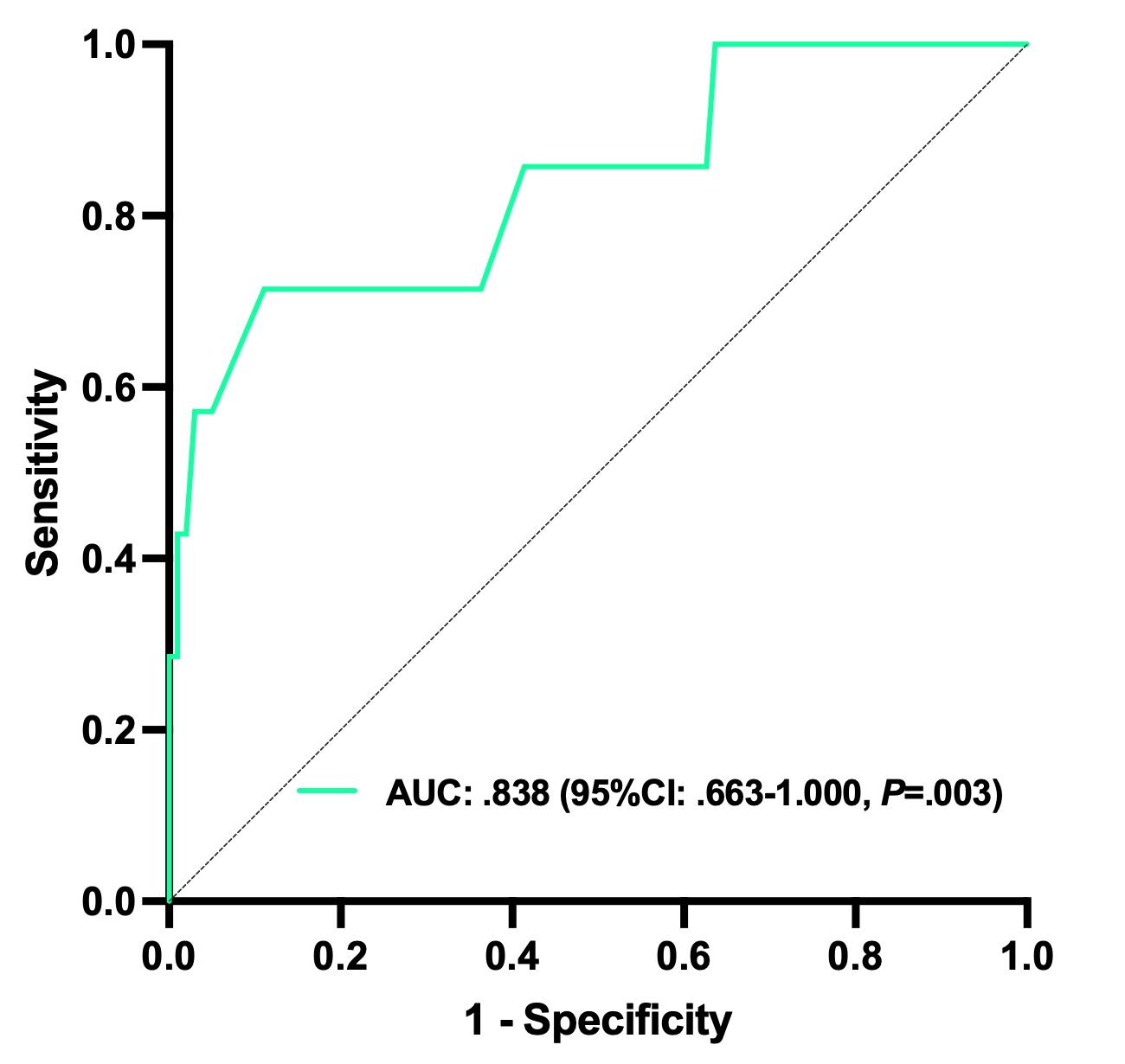

Supplement: Supplementary file 1 — Appendix S1. [file ECI-56-e70190-s001.zip › eci70190-sup-0002-FigureS2@Fig S2.tiff]

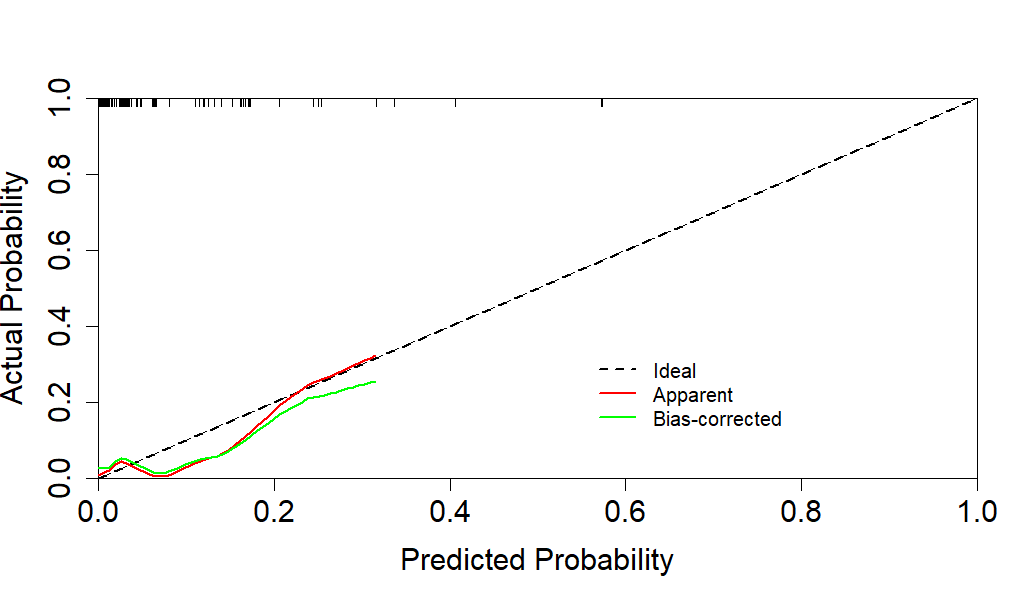

Supplement: Supplementary file 1 — Appendix S1. [file ECI-56-e70190-s001.zip › eci70190-sup-0003-FigureS3@Fig S3.tiff]

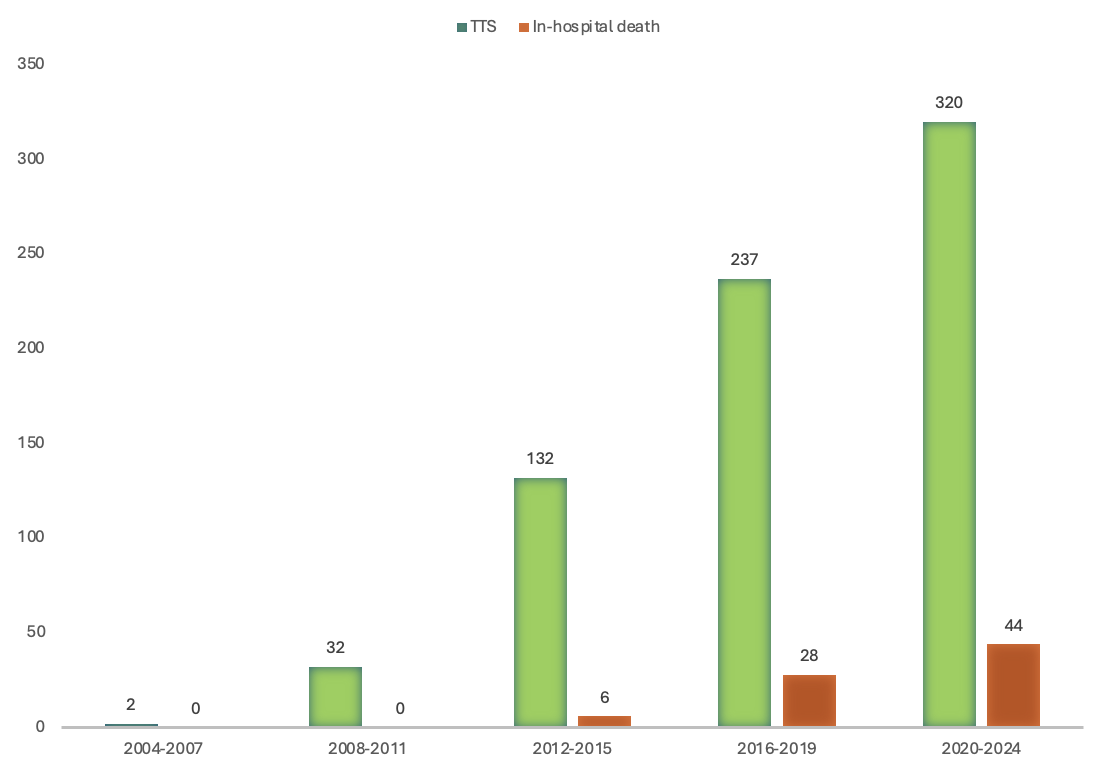

Supplement: Supplementary file 1 — Appendix S1. [file ECI-56-e70190-s001.zip › eci70190-sup-0004-FigureS4@Fig S4.tiff]

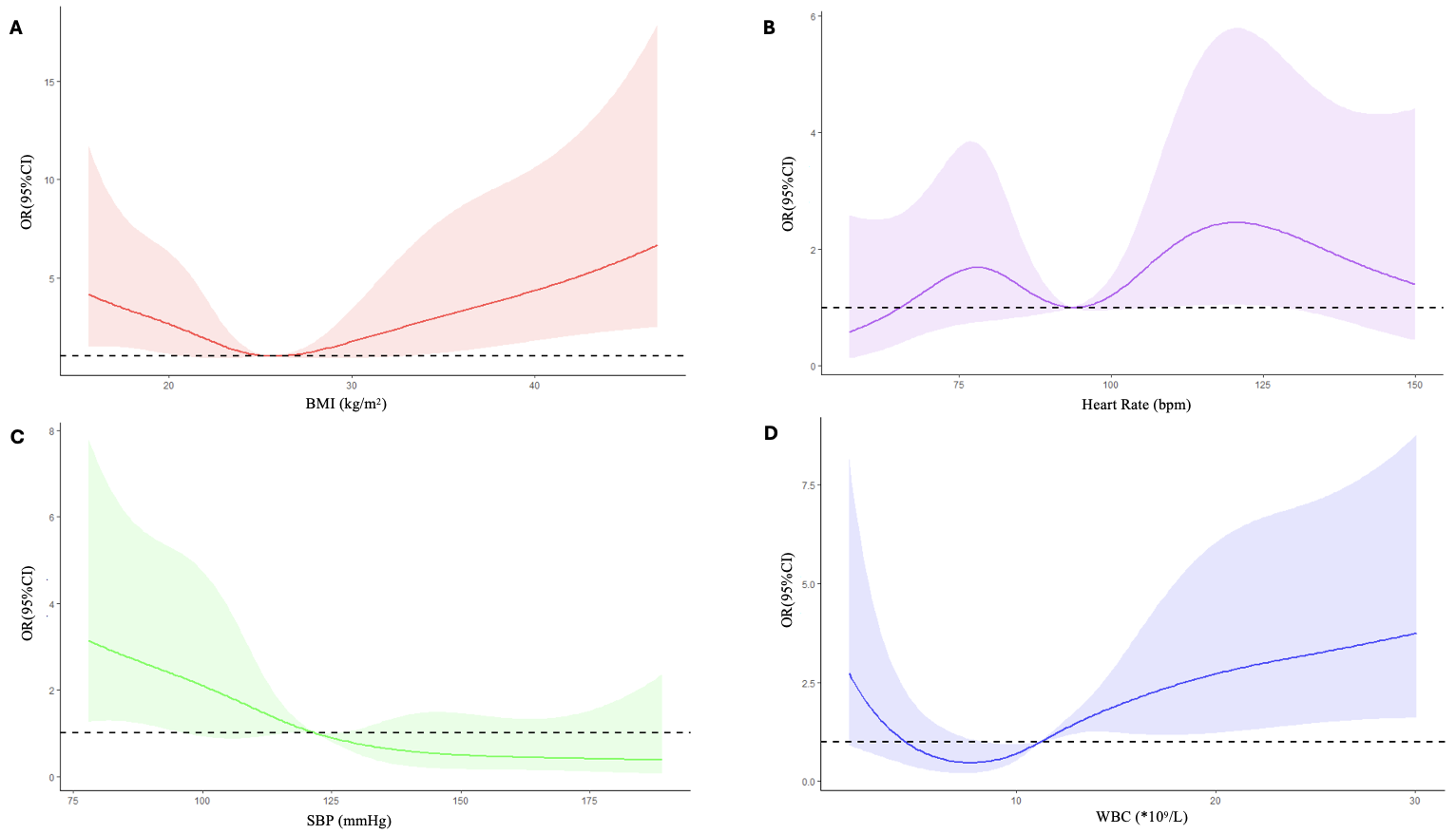

Supplement: Supplementary file 1 — Appendix S1. [file ECI-56-e70190-s001.zip › eci70190-sup-0001-FigureS1@Fig S1.tiff]
